# An Updated Reappraisal of Dupilumab in Children and Adolescents with Severe Asthma

**DOI:** 10.3390/children11070843

**Published:** 2024-07-11

**Authors:** Gian Luigi Marseglia, Amelia Licari, Maria Angela Tosca, Michele Miraglia del Giudice, Cristiana Indolfi, Giorgio Ciprandi

**Affiliations:** 1 Department of Clinical, Surgical, Diagnostic and Pediatric Sciences, University of Pavia, 27100 Pavia, Italy; gl.marseglia@smatteo.pv.it (G.L.M.); a.licari@smatteo.pv.it (A.L.); 2 Pediatric Clinic, Fondazione IRCCS Policlinico San Matteo, 27100 Pavia, Italy; 3 Allergy Center, IRCCS Istituto Giannina Gaslini, 16147 Genoa, Italy; mariangelatosca@gaslini.org; 4 Department of Woman, Child and General and Specialized Surgery, University of Campania “Luigi Vanvitelli”, 81100 Naples, Italy; michele.miragliadelgiudice@unicampania.it (M.M.d.G.); cristianaind@gmail.com (C.I.); 5 Allergy Clinic, Casa di Cura Villa Montallegro, 16145 Genoa, Italy

**Keywords:** severe asthma, type 2 inflammation, allergic asthma, dupilumab, phenotype

## Abstract

Severe asthma (SA) is still a demanding challenge in clinical practice. Type 2 inflammation is the most common phenotype in children and adolescents with SA. As a result, anti-inflammatory drugs, mainly corticosteroids (CSs), represent the first choice to reduce type 2 inflammation. However, SA patients may require high inhaled and oral CS doses to achieve and maintain asthma control. Some SA patients, despite the highest CS dosages, can even display uncontrolled asthma. Therefore, the biological era constituted a breakthrough in managing this condition. Dupilumab is a monoclonal antibody directed against the IL-4 receptor α-subunit (IL-4Rα), antagonizing against both IL-4 and IL-13, and has been approved for pediatric severe type 2 asthma. This review presents and discusses the most recent published studies on dupilumab in children and adolescents with SA. There is convincing evidence that dupilumab is a safe and effective option in managing SA as it can reduce asthma exacerbations, reduce CS use, and improve lung function, asthma control, and quality of life, also for caregivers. However, a thorough diagnostic pathway is mandatory, mainly concerning phenotyping. In fact, the ideal eligible candidate is a child or adolescent with a type 2 allergic phenotype.

## 1. Introduction

Asthma is a chronic medical condition that affects about 10% of the pediatric population [1]. The main clinical characteristics include episodes of dyspnea, coughing, chest tightness, and wheezing. Clinical features result from two leading pathophysiologic mechanisms: airway inflammation and hyperresponsiveness. Bronchial inflammation usually belongs to type 2 inflammation in childhood. Type 2 inflammation entails an expansion of type 2 immunocompetent cells, mainly including innate lymphocyte cells 2 and T helper 2. These cells, in turn, overexpress type 2 cytokines, such as interleukin-4 (IL-4), IL-5, and IL-13 [2]. These type 2 cytokines, on the one hand, switch the immunoglobulin production toward the E isotype. On the other hand, these type 2 cytokines, mainly IL-5, promote the production, infiltration, activation, and survival of eosinophils into bronchi [3]. Hyperresponsiveness sustains bronchial obstruction due to some stimuli: specific (allergens) or not specific (dry and cold air, pollutants, and smoke). Typically, children and adolescents experience asthma exacerbations (AEs) when exposed to high allergen concentrations and/or viral respiratory infections [4]. However, these exacerbations implicate a reversible bronchial obstruction. Interestingly, the main predictive factor for exacerbation is the number of previous ones [5]. Over time, some asthmatic subjects present tissue remodeling and progressive functional decline that result in fixed bronchial obstruction. Consequently, ideal asthma management should prevent asthma from worsening by reducing type 2 inflammation and reinforcing the immune system to avoid the negative impact of infections. In this regard, asthma treatment targets achieving and consolidating a state of good asthma control, i.e., few or no symptoms, use of relievers, and no limitation of personal activities.

Nevertheless, some children and adolescents present severe asthma (SA). The most recognized definition of severe asthma entails the deficient control of asthma symptoms, despite therapy with high doses of inhaled corticosteroids (ICSs) and long-acting β2-agonists (LABAs), the need for recurrent oral corticosteroids (OCSs) (level 4–5 GINA guidelines), or the loss of asthma control when reducing high-intensity treatment [6]. Fortunately, severe asthma is rare in childhood and adolescence, affecting <5% of asthmatics [7]. However, severe asthma significantly burdens the healthcare system and negatively impacts young patients and their families. Accordingly, children and adolescents with SA, mainly if asthma is uncontrolled, have frequent severe AE that, in turn, predisposes to impaired lung function and poor quality of life, and the use of high doses of CSs with the risk of adverse reactions [8]. These factors also increase the healthcare burden because they are associated with frequent unscheduled visits, emergency room visits, hospitalization admissions (up to intensive unit access), school absences, and loss of working days for parents [9]. Moreover, SA is a relevant risk factor for the later onset of chronic obstructive pulmonary disease (COPD) [10]. Considering these aspects, appropriate SA treatment, including AE prevention, is a mandatory target in clinical practice.

As inflammation is the leading cause of severe asthma, anti-inflammatory drugs are extensively used in asthma management. However, severe asthma treatment requires very high dosages of inhaled corticosteroids to control the disease. Notwithstanding, systemic corticosteroids, mainly oral, are frequently prescribed to achieve asthma control or treat asthma exacerbations. Unfortunately, some children and adolescents also require maintaining OCSs to control asthma. This excessive use of corticosteroids carries the risk of serious adverse events, especially in younger subjects. For this reason, new treatments have been developed. Biologic drugs represent a keystone in managing subjects with SA [11].

Presently, only three biologics have the indication for treating SA in children and adolescents: omalizumab, mepolizumab, and dupilumab [12]. All of them are targeted to contrast type 2 inflammation [13]. Omalizumab is a monoclonal antibody that antagonizes IgE, mepolizumab IL-5, and dupilumab contrasts the IL-4/IL-13 pathway. The present article concerns only dupilumab, so it presents and discusses the most recent studies on dupilumab use in pediatric asthma.

## 2. Dupilumab: Pharmacologic Profile

Dupilumab is a monoclonal antibody directed against the IL-4 receptor α-subunit (IL-4Ra), antagonizing against both IL-4 and IL-13, and has been approved for pediatric severe type 2 asthma [14]. As a result, dupilumab blocks the a subunit of the IL-4 receptor IL-4Rα, inhibiting the signal transduction pathways activated by IL-4 and IL-13. There are two main IL-4 receptors: type I and type II [15]. Type I consists of two sub-units: γ-chain associated with the Janus-family protein kinase 3 (JAK3) and IL-4Ra unit interacting with JAK1. Type II includes two sub-units: IL-4Ra associated with JAK1 and IL-13Ra1 acting with JAK2. In detail, dupilumab presents a plural mechanism of action, including the inhibition of IL-4 binding to IL-4Rα, the inhibition of the recruitment of γ-chain of the type 1 receptor to IL-4Rα chain, and the inhibition of the recruitment of the IL-4Rα to IL-13Rα1 for type II IL-4R [16]. IL-4Rα is ubiquitously expressed in innate and adaptive immune cells to promote the signaling of IL-4 and IL-13. T helper 2, innate lymphocyte cells 2, eosinophils, and mast cells are the primary sources of IL-4 and IL-13. IL-4 and IL-13 are prototypical type 2 cytokines driving type 2 inflammation. In particular, IL-4 promotes type 2 polarization and stimulates IgE production. IL-13 mostly favors bronchial hyperresponsiveness, eosinophilic airway inflammation, and tissue remodeling. Consequently, dupilumab interrupts the inflammatory cascade promoted by IL-4 and IL-13 and the progression of the type 2 pathway.

The present pediatric indication of dupilumab is for treating adolescents and children from six years of age with asthma. In particular, the European Medicine Agency (EMA) has approved the use of dupilumab in subjects suffering from type 2 SA characterized by a high peripheral eosinophil count and/or high levels of fractional exhaled nitric oxide (FeNO) associated with asthma symptoms that are not controlled by high-dose ICSs plus another controller drug in maintenance. The Global Initiative for Asthma (GINA) guidelines recommend the use of dupilumab in step 5 of treatment as an add-on strategy for patients older than 12 years with a high blood eosinophil count (>300/μL), high FeNO levels (>20 ppb), and uncontrolled asthma symptoms, despite high-dose ICSs associated with LABA or OCSs [17].

The dupilumab usable pharmaceutical form is a prefilled syringe for subcutaneous injection. The posology is initially 400 mg once per week, followed by 200 mg every fortnight. However, if patients have OCS-dependent SA associated with comorbidity, such as atopic dermatitis or chronic rhinosinusitis with nasal polyps, the initial dose should be 600 mg, followed by 300 mg on alternate weeks.

Regarding safety, the most common side effects include injection site reactions and eye and eyelid inflammation, including redness, swelling, itching, blurred vision, cold sores in the mouth or lips, rhinopharyngitis, and headaches [18]. Uncommonly, dupilumab may induce adverse reactions, including joint aches, eyelid swelling and inflammation, and allergic reactions, such as shortness of breath and wheezing [18].

Regarding the contraindications, hypersensitivity to any of dupilumab excipient dupilumab is the most relevant contraindication. The Food and Drug Administration (FDA) recommends caution only against using live virus vaccines in dupilumab recipients. However, no concrete data are available on the ability of live vaccines to trigger an immune response [19]. Also, the FDA suggests completing all age-appropriate immunizations before beginning dupilumab treatment. In addition, the FDA advises treating patients with a preexisting helminthic infection with anti-helminthic therapy before initiating dupilumab [19].

## 3. Dupilum: Clinical Evidence

Dupilumab has been extensively investigated in some randomized controlled trials (RCT) in adult patients with asthma. However, the most important trials conducted on pediatric patients include two primary studies: the phase III LIBERTY ASTHMA QUEST, involving adolescents, and the VOYAGE study, recruiting children aged 6 to 12 years [20,21]. The LIBERTY ASTHMA QUEST study included and randomly assigned 1902 patients (12 years of age or older) with uncontrolled asthma to receive (in a 2:2:1:1 ratio) add-on subcutaneous dupilumab 200 or 300 mg every 2 weeks or matched-volume placebos for one year [20]. The annualized rate of severe asthma exacerbations and the absolute change in the first trimester of the pre-bronchodilation forced expiratory volume was 1 s (FEV_1_) in the overall trial population. The results showed that the patients who received dupilumab had significantly lower rates of severe asthma exacerbation than the control and better lung function and asthma control. Notably, patients with higher levels of eosinophils at baseline had the highest response.

The VOYAGE study included 408 children (6 and 11 years) with uncontrolled moderate-to-severe asthma [21]. Children received a subcutaneous injection of dupilumab (100 mg for ≤30 kg/bw and 200 mg for >30 kg/bw) or matched placebo every 2 weeks for 24 weeks. The primary end point was the annualized rate of severe asthma exacerbations. Add-on dupilumab induced fewer asthma exacerbations and better lung function and asthma control than the placebo.

Other studies have been performed and evaluated by some meta-analyses. Two relatively recent reviews reported studies published up until 2022 [14,22]. Therefore, the present review will summarize the meta-analyses and up to date investigations, mainly focusing on the QUEST and VOYAGE studies.

### 3.1. Meta-Analyses

Zaazouee and colleagues provided a systematic review and meta-analysis on dupilumab’s efficacy and safety in patients with moderate to severe asthma [23]. This investigation evaluated the studies published by January 2022 and selected 13 RCTs for the systematic review and 9 for the meta-analysis; globally, 4482 patients participated in these trials. Seven studies recruited pediatric patients. The findings showed that dupilumab significantly improved lung function (e.g., FEV_1_), asthma control questionnaire (ACQ) scores, FeNO levels, and IgE values at 12 and 24 weeks. Dupilumab, however, increased the blood eosinophil count at the same time. Finally, adverse reactions to dupilumab were minimal and comparable to the placebo.

Charles and coworkers performed a systematic review and meta-analysis of the real-world efficacy of some biologics, including dupilumab, for severe asthma [24]. This study selected 22 real-world studies on anti-cytokine biologics, but only one study concerned dupilumab, which was not considered in the meta-analysis. Anyway, the results for dupilumab showed a reduction in the Δ annualized AE and ΔOCS dosage rates.

Akenroye and others performed a Bayesian network meta-analysis to compare the efficacy of mepolizumab, benralizumab, and dupilumab in eosinophilic asthma, considering papers published up until 2021 [25]. The analysis selected eight RCTs, two of which were concerned with dupilumab. The investigators stratified the population into two subgroups, considering patients with ≥300 blood eosinophils per microliter or less. In patients with high blood eosinophils, dupilumab, compared with the placebo, significantly reduced the AE rates, improved FEV_1_, and reduced ACQ. The results could have been more relevant in patients with a lower eosinophil count. The authors concluded that these three biologics produced comparable outcomes on AE, lung function, and asthma control.

The same research team conducted another Bayesian network meta-analysis, including tezelepumab, in eosinophilic asthma, considering studies published up until August 2022 [26]. The analysis evaluated 10 RCTs. The two dupilumab studies showed a >99% probability of reducing AE by >50% compared with the placebo. In addition, dupilumab had a possibility of increasing prebronchodilator FEV1 by >100 mL above the placebo of 1.00. Dupilumab, compared with mepolizumab, had a >90% chance of improving FEV_1_ by >50 mL, but none of the differences between biologics exceeded 100 mL.

Phinyo et al. performed a systematic review and network meta-analysis on the efficacy and safety of some biologics for OCS-dependent asthma [27]. The analysis included seven RCTs recruiting 1052 patients with OCS-dependent asthma. Benralizumab, administered every 4 or 8 weeks, dupilumab, and mepolizumab reduced the OCS dose compared with the placebo (without inter-group difference), whereas tralokinumab, tezepelumab, and subcutaneous reslizumab were ineffective. Interestingly, a high blood eosinophil count at baseline benefited from anti-IL-5 biologics, whereas high FeNO levels favored dupilumab (regardless of initial blood eosinophil counts). Adverse events between biologics and the placebo were comparable, except for eosinophilia induced by dupilumab. The authors concluded that a thorough assessment of biomarkers allows for a precise selection of the appropriate biologic. In other words, dupilumab should be the first choice for patients with high FeNO levels, independent of the eosinophils level.

### 3.2. QUEST Study

The QUEST study was a phase III trial of 52 weeks and included 107 adolescents [20]. This trial was the first to explore the efficacy of dupilumab in adolescents. It was preceded by a phase III 24-week treatment trial (LIBERTY ASTHMA VENTURE), including only three adolescents [28]. The QUEST study demonstrated a reduced AE rate and improved lung function and symptom control in the entire recruited population [20]. Remarkably, patients with type 2 inflammation, detected by blood eosinophil count or FeNO, reported the most remarkable effects [20,28].

A post hoc analysis successfully investigated efficacy in adolescents [29]. This analysis included 127 adolescents: 34 treated with 200 mg dupilumab, 34 with 300 mg dupilumab every two weeks, and 39 with a placebo. Dupilumab (200 mg every two weeks) significantly reduced the severe AE rate compared with the placebo (0.19 versus 0.36 annualized rate). Conversely, high-dose dupilumab (300 mg every two weeks) increased the risk of AE by 13% in exacerbations in comparison with the placebo (0.37 vs. 0.33). This increased risk also persisted after stratifying adolescents by type 2 phenotype (blood eosinophils ≥ 150 cells/μL or FeNO ≥ 20 ppb). An imbalanced number of AEs between subgroups in the preceding year was a possible explanation. Moreover, both dupilumab dosages (200 mg and 300 mg) improved lung function upon 12 weeks of treatment, but did not significantly reduce symptom severity.

Pavord and colleagues performed another post hoc analysis of the QUEST study to evaluate the baseline FeNO value as an independent predictive factor of the dupilumab response [30]. The analysis stratified the patients into three subgroups considering the baseline FeNO levels: <25 ppb, between 25 and 50 ppb, and >50 ppb. Patients with the highest baseline FeNO experienced less AE and had higher FEV_1_ values after dupilumab treatment. The results were independent of peripheral eosinophil counts and other clinical characteristics.

### 3.3. VOYAGE Study

Dupilumab approval was also extended to children older than six years, which was made possible by the results of the VOYAGE study [21]. This phase III RCT assessed the efficacy of add-on dupilumab in 408 children (6–11 years) suffering from uncontrolled moderate-to-severe asthma. Most children (86%) had the type 2 asthma phenotype as documented by the peripheral eosinophil count (≥150 cells/μL) or FeNO (≥20 ppb). Dupilumab or matched placebo was administered bimonthly for 52 weeks.

The primary outcome of the VOYAGE study was the annualized rate of severe AE, defined as asthma-dependent OCS use for at least three days, hospitalization, or emergency department (ED) visits leading to OCS use [21]. The findings evidenced that dupilumab reduced the risk of severe AE in the study population, as indicated by the rate ratio (RR) of dupilumab versus placebo of 0.46.

Considering the dupilumab mechanism of action, the effect size was highest in children presenting the most pronounced type 2 phenotype, characterized by peripheral eosinophils of ≥300 cells/μL. Interestingly, 63% of all participants in the trial had ≥300 cells/μL.

The secondary outcomes were lung function improvement upon 12 weeks of treatment and a change in the asthma control score upon 24 weeks. The results showed that dupilumab treatment significantly improved FEV_1_ values and asthma control, measured by ACQ-7. Consistently, the secondary outcomes were higher in children with evident type 2 phenotype.

In addition, the VOYAGE study also considered several secondary outcomes that generated a wealth of data that have been the subject of several publications.

A post hoc analysis investigated biomarkers and pharmacokinetics [31]. The VOYAGE study included the measurement of type 2 biomarkers, including serum total IgE (IU/mL), serum thymus and activation-regulated chemokine (TARC) (ng/L), blood eosinophil count (cells/μL), and FeNO (ppb) over the 52 weeks of treatment. Both dupilumab regimens significantly reduced all type 2 biomarkers at the end of the trial.

Regarding pharmacokinetics, the dupilumab serum levels reached a steady state by week 12. The analysis stratified the selected children into two subgroups according to the schedule: children receiving 100 mg or 200 mg bimonthly; the mean concentrations were 51.2 mg/L or 79.4 mg/L, respectively. These concentrations are consistent with those observed in adults and adolescents, ranging between 29 and 80 mg/L.

A further publication used validated instruments to analyze two outcomes: asthma control and health-related quality of life (HRQoL) [32]. A primary outcome of this derived study was the asthma control change from baseline using the Asthma Control Questionnaire 7–Interviewer-Administered (ACQ-7-IA). The analysis aimed to identify children achieving a clinically meaningful response of ≥0.5 points and calculated the proportion of children achieving well-controlled asthma or better (ACQ-7-IA ≤0.75 points). The other primary outcome was to assess the treatment effect on patients’ and caregivers’ quality of life. Investigators administered to children the Standardised Paediatric Asthma Quality of Life Questionnaire–Interviewer-Administered (PAQLQ(S)-IA) and the Paediatric Rhinoconjunctivitis Quality of Life Questionnaire–Interviewer-Administered (PRQLQ-IA) for asthma and allergic rhinitis, respectively. The Paediatric Asthma Caregiver’s Quality of Life Questionnaire (PACQLQ) was administered to the caregivers.

The findings showed that dupilumab, compared with the placebo, significantly improved the asthma control measured with ACQ-7-IA by week 4 and, overall, showed marked improvements through week 52. In particular, most dupilumab-treated children had well-controlled asthma at weeks 24 and 52.

Regarding QoL, children and their caregivers provided significantly improved PAQLQ(S)-IA and PACQLQ scores by week 52. Therefore, the authors concluded that dupilumab induced quick and marked improvement of asthma control and quality of life in children aged 6–11 years with moderate-to-severe type 2 asthma and also ameliorated the caregivers’ QoL.

A parallel analysis regarded the study of lung function [33]. Also, this study’s evaluated population included children with type 2 inflammation (350) alone. The spirometry parameters were FEV_1_, FVC, FEV_1_/FVC, and FEF_25–75_, measured before the bronchodilation test and post-bronchodilator FEV_1_, evaluated at baseline and at the end of the trial. 

As regards the pre-bronchodilator spirometry, dupilumab treatment significantly increased FEV_1_; interestingly, the most evident effect was observed in children with initial z-score values < 1.64. After bronchodilation, dupilumab significantly increased FEV_1_, both considering the absolute values and z-scores. Notably, the response to bronchodilation was reduced by dupilumab.

Consistently, dupilumab treatment significantly improved FVC, FEV_1_/FVC, and FEF_25–75_ both pre-bronchodilation and post-bronchodilation.

The EXCURSION study was an open-label extension of the VOYAGE trial [34]. This study assessed the long-term safety and efficacy of dupilumab in 365 children with moderate-to-severe asthma. The 240 children, initially treated with dupilumab, conserved the add-on dupilumab regimen, and the dose was adjusted for their body weight (100 mg for ≤30 kg or 200 mg for >30 kg every two weeks), maintaining the same schedule; the study lasted for 52 weeks. In addition, 125 children, previously allocated to the placebo group, started dupilumab treatment with the same schedule. The primary endpoint was the number and proportion of children without any treatment-emergent adverse event (TEAE) throughout the 52-week study duration. The results demonstrated that, in the global population, 232 (63.6%) children experienced at least one TEAE (without difference between groups). The most frequently reported TEAEs were rhinopharyngitis, pharyngitis, and upper respiratory tract infections.

The secondary endpoints examined in EXCURSION included efficacy outcomes on severe exacerbation, lung function, and type 2 inflammation biomarkers. The findings confirmed the results observed in the parental study VOYAGE for all parameters and also in the previous placebo group.

Papadopoulos and colleagues performed a post hoc analysis of the VOYAGE study considering the effect of the presence or absence of allergic asthma, defined by baseline serum total IgE values ≥30 IU/mL and ≥ one perennial aeroallergen-specific IgE ≥ 0.35 kU/mL [35]. This analysis considered annualized severe exacerbation rates, lung function (pre-bronchodilator FEV_1_, evaluated as absolute value and predicted value), and asthma control (measured by ACQ-7 over 12 months). Children with allergic asthma were 261 (74.5%). Dupilumab treatment reduced AE compared with the placebo in allergic children (relative RR 0.24 vs. 0.62) and non-allergic children (0.39 vs. 0.8). A significant improvement in lung function was observed in allergic children, but only numerical in non-allergic ones. Finally, asthma control was significantly improved only in allergic patients. Based on these findings, the authors concluded that the effect of dupilumab on AE was irrespective of allergic evidence. In contrast, effects on lung function and mostly on asthma control were evident in allergic children alone.

Notably, it has to be underlined that the EXCURSION study is the first two-year study of an injectable biologic investigated in children with severe asthma, as precisely noted [36]. Moreover, the authors of this comment suggested that dupilumab should be the first-line biologic in children 6–11 years with severe type 2 asthma as mepolizumab, an anti-IL-5 biologic, slightly reduced asthma attacks in a recent phase III study [37].

### 3.4. Other Recent Studies

Other recent studies are summarized in Table 1. Monzio Compagnoni and coworkers conducted a real-life study on a population-based cohort of 46 children with difficult-to-control asthma [38]. The children aged 6–11 years were undergoing treatment with dupilumab [4], mepolizumab [5], or omalizumab [37]. The study analyzed the mean one-year healthcare costs and resource utilization, comparing data from one year before and the year after the treatment’s initiation (follow-up period). The biologic treatment reduced exacerbation-related hospitalization by 75% and emergency room access by 85.7%. Moreover, biologics reduced OCSs, short-acting β2-agonists (SABAs), and fixed associations of SABAs and ICSs. The discontinuation rate was modest, reaching 6.5%. In addition, the overall healthcare costs increased because of the high costs of biologics. However, the hospital admission-related costs significantly diminished.

Buendia and Patino reported conflicting outcomes when estimating the dupilumab cost–utility in Colombia using a Markov-type model [39]. The estimated cost–utility and health outcomes of dupilumab plus standard of care were compared with the cost–utility and health outcomes of the standard of care alone. The analysis considered a simulated cohort of children with persistent asthma treated over six years. The results showed that the estimated quality-adjusted life-years (QALYs) per patient were 0.85 with add-on dupilumab and 0.84 with standard of care. The dupilumab total mean discounted cost per patient per cycle was USD 379, whereas that for standard of care was USD 19. The incremental cost–effectiveness ratio estimated was USD 24,660 per QALY. Therefore, the authors concluded that dupilumab was not cost-effective in Colombian 6–11-year-old children with severe type 2 asthma. However, it should be noted that this study was conducted in Colombia, a country with relevant socio-economic problems, and used a simulated cohort of patients. Therefore, the results should be cautiously evaluated. 

Dinardo and colleagues reported the case of a 15-year-old subject with severe asthma presenting frequent AE and allergic rhinitis comorbidity [40]. This case report showed that this adolescent reported a significant improvement in FEV_1_, asthma control (measured by ACQ-7) and reductions in the ICS dose and FeNO levels after only two weeks of treatment. Thus, this report outlined the fast onset of action provided by dupilumab. The authors commented that a rapid effect could increase confidence in the treatment and improve adherence to prescribed therapy. This preliminary experience was confirmed by a series of five adolescents with severe uncontrolled asthma and treated with add-on dupilumab for 24 weeks [41]. The findings showed that dupilumab quickly improved QoL by the fourth week, and four adolescents halved their ICS doses. These outcomes were maintained until the 24th week. Notably, no patients had any AE during the study period.

Votto and collaborators retrospectively evaluated a new set of core outcome measures for asthma (COMSA) in 31 children (6–15 years old) with severe asthma. They treated the patients with omalizumab [19], mepolizumab [6], or dupilumab [6] as an add-on treatment [42]. The efficacy parameters included pre-bronchodilator FEV_1_, FEF_25–75_, AE, quality of life, and asthma control (evaluated by GINA grading and ACT). The results demonstrated that these biologics significantly reduced the absolute values of FEV_1_, FEF_25–75_, AE, OCS use, quality of life, and ACT scores. This real-life study confirmed the efficacy of biologics, including dupilumab, in children with severe asthma and the suitability of COMSA parameters in the pediatric setting.

Finally, an RCT (PANDA study) is currently ongoing to investigate the effect of add-on dupilumab for 12 months on AE in urban children and adolescents aged 6–17 years with type-2 asthma and who are exacerbation-prone [43].

Managing children and adolescents with severe asthma is a compelling challenge in daily practice. The advent of biologics represents an outstanding breakthrough in SA therapy [44]. In particular, the model of Precision Medicine commenced a new management approach based on the definition of specific phenotypes and endotypes [45]. Moreover, Precision Medicine is the natural premise for Personalized Medicine [46]. Namely, tailored biologics could effectively and safely modify the natural history of SA.

**Figure 1:**
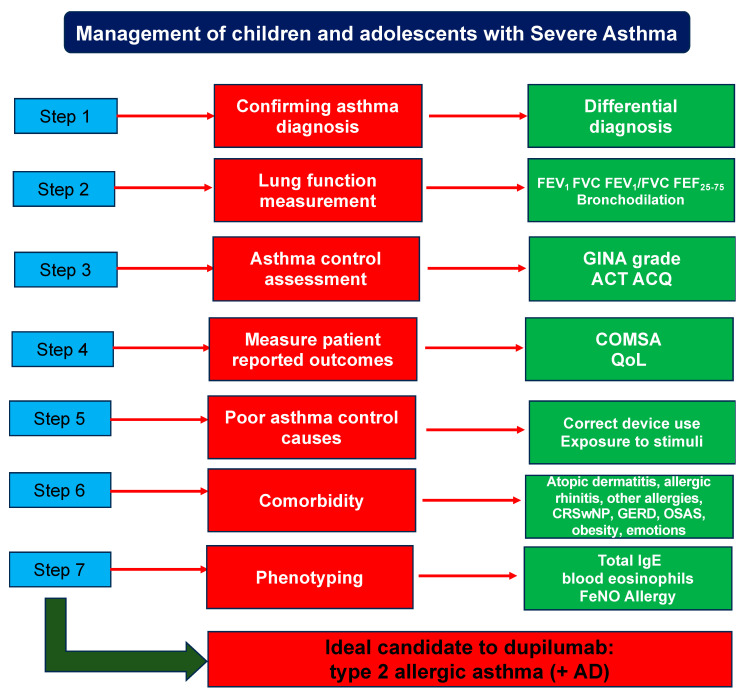
Pragmatic management of children and adolescents with severe asthma. ACT: asthma control test; ACQ: asthma control questionnaire; COSMA: core outcomes measures for asthma; QoL: quality of life; CRSwNP: chronic rhinosinusitis with nasal polyps; GERD: gastroesophageal reflux disease; OSAS: obstructive sleep apnea syndrome; FeNO: fractional exhaled nitric oxide: AD: atopic dermatitis.

These concepts are basic for proper management and optimal responses to therapy. In this regard, the correct and appropriate approach to managing children and adolescents with SA consists of a stepwise assessment of each patient’s clinical, functional, and biological characteristics, as schematized in Figure 1 [47]. Clinicians should confirm the asthma diagnosis, measure lung function, score the asthma control grade, measure the patient’s reported outcomes, understand the causes of poor asthma control, and identify (if any) comorbidities and the phenotype of each patient. 

Asthma diagnosis requires a thorough evaluation considering the clinical history, demonstration of bronchial obstruction and its reversibility, and exclusion of other confounding respiratory diseases.

Asthma control should be measured according to validated criteria recommended by GINA guidelines [17]. In addition, asthma control may be measured using consolidated questionnaires, including ACT (C-ACT) and ACQ. 

An accurate search of causes of poor asthma control should include the assessment of adherence to inhalation techniques, exposure to allergens and/or tobacco smoke, and therapy compliance. Doctors should consider the presence of comorbidity, mainly including atopic dermatitis, allergic rhinitis, other allergies, chronic rhinosinusitis, gastroesophageal reflux, obstructive apnea, obesity, and emotional disorders, as outlined in the GINA guidelines version 2024 [17].

Phenotyping is the keystone to choosing the most suitable biologic for each patient. In this regard, biomarkers allow correct phenotyping of patients. Unfortunately, few biomarkers are still available in clinical practice to correctly phenotype patients with SA and type 2 inflammation, including the serum total IgE, peripheral eosinophils, FeNO, and allergy [48]. In particular, allergy, such as allergic asthma, is defined by the consistency between history and sensitization, such as the production of allergen-specific IgE [49]. In other words, a subject is defined as allergic when experiencing symptoms immediately after inhaling the sensitizing allergen [50]. It has to be underscored that sensitization alone is not sufficient to diagnose allergic asthma.

At the end of this diagnostic pathway, it is possible to choose the ideal biologic. Dupilumab is recognized as an optimal eligible candidate for a child with an allergic type 2 phenotype. Namely, the reported studies underlined the relevance of type 2 phenotype and allergic mechanisms as predictive factors of optimal response

## 4. Conclusive Remarks

Dupilumab is a safe and effective biologic indicated in 6–11-year-old children and adolescents with severe asthma. After precise and thorough framing, the ideal candidate for dupilumab ideally should be a patient with SA characterized by the type 2 allergic phenotype; in addition, as dupilumab also has an indication for atopic dermatitis, children with this condition and SA could optimally take advantage of this biological drug. However, greater attention should be paid to the side effects of blocking the IL-4/IL-13 pathway, since potential side effects are frequently questioned in pediatric care. In this regard, there were observations of increased eosinophilia, infections, and conjunctivitis during placebo-controlled trials using dupilumab. In this regard, the large experience for children with atopic dermatitis provided relevant information, analytically reported and discussed in two outstanding reviews and one real-world meta-analysis [51,52,53].

## Figures and Tables

**Table 1:** Dupilumab: a pragmatic approach in clinical practice.

Study (Ref)	Age (Years)	Design	Number Patients	Duration	Outcomes
QUEST study	12–17				
Castro et al. [20]		DB-PC	127	52 w	Reduced AEs Improved lung function
Pavord et al. [30]		DB-PC	127	12 w	High basal FeNO predicted response
VOYAGE study	6–11				
Bacharier et al. [21]		DB-PC	408	52 w	Reduced AEs (hospitalization, ED visits) Increased FEV_1_ Improved asthma control
Fiocchi et al. [31]		DB-PC	408	52 w	Improved QoL and ACQ-7
Jackson et al. [32]		DB-PC	350	52 w	Pharmacokinetics Reduced total IgE, TARC, blood eosinophils, and FeNO
Bacharier et al. [33]		DB-PC	350	52 w	Improved lung function
Bacharier et al. [34]		Open	365	52 w	TEAE Reduced AEs and type 2 biomarkers
Papadopoulos et al. [35]		DB-PC	350	52 w	Allergic children better responded
Recent studies					
Monzio Compagnoni et al. [38]	6–11	Retrospective	46	1 year	Reduced AEs and drug use Reduced costs
Buendia and Patino [39]	6–11	Markov-type model	Simulated cohort	6 years	Dupilumab increased costs
Dinardo et al. [40]	15	Case report	1	2 w	Improved FEV_1_; asthma control reduced ICS dose and FeNO
Indolfi et al. [41]	12–17	Case report	5	24 w	Improved QoL, reduced ICS dose, no AEs
Votto et al. [42]	6–15	Retrospective	31	1 y	Improved lung function, asthma control, and QoL, and reduced AEs
PANDA study [43]	6–17	DB-PC		52 w	Ongoing

DB-PC: double-blind placebo-controlled; w: weeks; AEs: asthma exacerbations; FeNO: fractional exhaled nitric oxide; ED: emergency department; TARC: thymus and activation-regulated chemokine; QoL: quality of life; ACQ-7: asthma control questionnaire with 7 items; TEAE: any treatment-emergent adverse event.
